# Association of HER1 and HER2 Gene Variants in the Predisposition of Colorectal Cancer

**DOI:** 10.1155/2021/6180337

**Published:** 2021-10-21

**Authors:** Ibrahim O. Alanazi, Jilani Purusottapatnam Shaik, Narasimha Reddy Parine, Nahla A. Azzam, Othman Alharbi, Yousef M. Hawsawi, Atif Abdulwahab A. Oyouni, Osama M. Al-Amer, Faisal Alzahrani, Majid A. Almadi, Abdulrahman M. Aljebreen, Mohammad Saud Alanazi, Zahid Khan

**Affiliations:** ^1^The National Center for Biotechnology, King Abdulaziz City for Science and Technology, P.O. Box 6086, Riyadh 11442, Saudi Arabia; ^2^Genome Research Chair, Department of Biochemistry, College of Science, King Saud University, Riyadh, Saudi Arabia; ^3^College of Medicine, King Saud University, Riyadh, Saudi Arabia; ^4^Division of Gastroenterology, King Khalid University Hospital, King Saud University, Riyadh, Saudi Arabia; ^5^Research Center, King Faisal Specialist Hospital and Research Center, MBC-J04, PO Box 40047, Jeddah 21499, Saudi Arabia; ^6^College of Medicine, Alfaisal University, P.O. Box 50927, Riyadh 11533, Saudi Arabia; ^7^Department of Biology, Faculty of Sciences, University of Tabuk, Tabuk, Saudi Arabia; ^8^Genome and Biotechnology Unit, Faculty of Sciences, University of Tabuk, Tabuk, Saudi Arabia; ^9^Department of Medical Laboratory Technology, Faculty of Applied Medical Sciences, University of Tabuk, Tabuk 71491, Saudi Arabia; ^10^Department of Biochemistry, Faculty of Science, Embryonic Stem Cells Unit, King Fahd Medical Research Center, King Abdulaziz University, Jeddah 21589, Saudi Arabia

## Abstract

**Background:**

Colorectal cancer (CRC) is a major health concern worldwide. A series of sequential accumulation of genetic and epigenetic changes are responsible for the initiation and progression of diseases via the normal > adenoma > carcinoma sequence. Genetic variants in crucial cancer-causing genes are known to mediate the risk of cancer.

**Objective:**

In this case-control study, we examined single nucleotide polymorphism (SNP) in HER1 (rs763317 and rs3752651) and HER2 (rs1136201 and rs1058808) genes to assess their role in the susceptibility of CRC in a Saudi population.

**Methods:**

TaqMan allelic discrimination assay was utilized to identify the genotypes in 163 normal and 143 CRC patients.

**Results:**

In the overall analysis, the rs3752651 and rs1136201 were significantly associated with the risk of CRC. Although none of the examined SNPs had any impact on the age at which CRC was diagnosed, interestingly, three SNPs showed a significant association based on gender. The rs3752651 conferred significant protection only in men, whereas rs1136201 diminished the risk and rs1058808 considerably increased the susceptibility of CRC only in women.

**Conclusions:**

Our result suggests that these SNPs in HER1 and HER2 after validation in larger cohorts of different ethnicities may be utilized as genetic screening markers for predicting colorectal cancer predisposition.

## 1. Introduction

Colorectal cancer (CRC) is one of the leading causes of cancer-related mortality and morbidity around the world [1]. As per gender segregation, it is the third commonly diagnosed cancer in men and ranks second in women [2]. In 2018, the estimated total number of new cases was 1,849,518 while the number of deaths was 880,792 [3]. The incidence and mortality of CRC in men are approximately 20% higher than in women. In Saudi Arabia, both the genders combined, CRC is the second most common cancer with an estimated incidence of 3564 (14.6%) cases and mortality of 1603 (15.2%) in the year 2018 [3].

Colorectal tumorigenesis is a multistep process involving accumulation of mutations in oncogenes and tumor suppressor genes leading to the sequential transformation of the colorectal normal epithelium to adenoma followed by invasive and metastatic tumor [4]. Various risk factors including ethnicity, environment, and genetics have been attributed towards the predisposition of CRC [5]. SNPs are considered to be the main source of genetic variation which accounts for about 90 percent of all human genetic variations occurring roughly every 100–300 bases [6]. Most SNPs are usually located in noncoding regions, and their distribution is not under selective pressure [7]. However, SNPs occurring in coding sequences of genes may lead to protein expression changes that directly contribute to a disease phenotype [8]. The location of these SNPs near genes that are involved in a crucial role such as gene expression and regulation, signal transduction, and the control of cell cycle may affect the function of such genes. An important molecular mechanism involved in the development and the progression of CRC is the mitogen-activated protein kinase (MAPK) cascade downstream of the epidermal growth factor receptor (EGFR) [9].

The EGFR also known as human epidermal growth factor receptor 1 (HER1) belongs to the ErbB family of transmembrane protein receptors that function as a receptor tyrosine kinase (RTK). The ErbB family consists of four members including ErbB-1 (HER1/EGFR), ErbB-2 (HER2), ErbB-3 (HER3), and ErbB-4 (HER4) [9]. Previous studies have reported that HER1 and HER2 gene polymorphisms are associated with several malignancies such as colon [10], breast [11, 12], and lung cancers [13]. Additionally, HER2 polymorphism has also been linked with higher expression of the protein in breast and ovarian cancers [14, 15].

Large efforts are being made in identifying trustworthy biomarkers for early detection of human malignant cancers. Genetic variation analysis such as SNPs can be used for predicting novel diagnostic and prognostic genetic markers. In this study, we examine the effect of four SNPs, rs763317 and rs3752651 located in HER1 and rs1058808 and rs1136201 located in HER2, on CRC susceptibility in Saudi Arabian patients by comparing the genotypic and allelic distribution of these SNPs in cancer cases to that of healthy subjects.

## 2. Materials and Methods

### 2.1. Sampling and DNA Isolation

Colorectal cancer patients (*n* = 143) included all ages (median age = 58 years) and histopathologically confirmed cases of the disease. Approximately 3 ml of blood samples from colorectal cancer patients attending the endoscopy department of King Khalid University Hospital, King Saud University, was collected in ethylenediaminetetraacetic acid- (EDTA-) containing vacutainers prior to therapy. Additionally, age- and gender-matched normal subjects with no history of cancer were recruited as controls. The study was reviewed and approved by the institutional review board of College of Medicine, King Saud University (Project approval No. E-12-596). Written informed consent was obtained from all the patients. Genomic DNA was isolated from peripheral blood cells using the QIAmp DNA blood mini kit (Qiagen, USA) according to manufacturer's instructions. Quantitation and purity of DNA were examined on a NanoDrop 8000 spectrophotometer (Thermo Scientific, USA).

### 2.2. Genotyping

Based on previous studies, four SNPs in HER1, rs763317 and rs3752651, and HER2, rs1058808 and rs1136201, were selected for this study [10, 16–19]. TaqMan allelic discrimination assays (Thermo Fisher Scientific, USA) were used for genotyping colorectal cancer and control peripheral blood DNA samples for these SNPs. The TaqMan assay ID and number of samples for which genotype calls were obtained for each SNP are as follows: rs763317 (assay ID = C___2310200_10, cancer = 140, control = 149), rs3752651 (assay ID = C____326500_10, cancer = 143, control = 163), rs1058808 (assay ID = C___1551672_20, cancer = 139, control = 160), and rs1136201 (assay ID = C___7452451_1_, cancer = 141, control = 162). Each reaction contained 20 ng of DNA, 5.0 *μ*L of 2X TaqMan genotyping Master Mix (Applied Biosystems, USA), and 0.25 *μ*L of 40X TaqMan assay in a total volume of 10 *μ*L. Genotyping experiments were conducted by endpoint reading on the QuantStudio 7 Flex Real-Time PCR machine (Applied Biosystems, USA), and genotype calls were made by TaqMan Genotyper Software version 1.6.

### 2.3. Statistical Analyses

Genotype and allele frequencies of polymorphic variants examined in HER1 and HER2 genes were determined, and tests for association and Hardy–Weinberg equilibrium were performed on a web-based tool at https://ihg.helmholtz-muenchen.de/cgi-bin/hw/hwa1.pl. For each SNP, odds ratios, confidence intervals, chi-square, and significance were calculated to assess the existence of an association between the SNP and colorectal cancer. Statistical significance was kept at a *p* value of ≤0.05. Bonferroni correction was used for multiple comparison with an *α* = 0.0125 considered as significant.

## 3. Results

In the present study, the genotypes of four germline SNPs residing in HER1, rs763317 and rs3752651, and HER2, rs1058808 and rs1136201, were evaluated in order to determine their association with colorectal cancer risk in Saudi Arabian population. The rs763317 and rs3752651 are located in the intron of HER1, whereas the rs1058808 (P1170A) and rs1136201 (Ile655Val) are nonsynonymous coding SNPs in HER2.

The distribution of genotypes of these SNPs in both the control and cancer groups was examined for Hardy–Weinberg equilibrium (HWE). All the SNPs followed Hardy–Weinberg equilibrium except rs3752651 (*p*=0.045373) in cancers (Table 1). A high rate of consanguinity in the Saudi Arabian population could be a probable cause for this lack of Hardy–Weinberg equilibrium [20].

### 3.1. Association of HER1 and HER2 Variants with Colorectal Cancer Risk

The distributions of genotype and allele frequencies of the chosen SNPs within the HER1 and HER2 genes and their association with colorectal cancer were evaluated between both colorectal cancer patients and healthy individuals (Table 2). The rs3752651 within HER1 showed a statistically significant association with colorectal cancer susceptibility. In the control group, the frequency of the CC genotype of rs3752651 was detected at 4%, whereas none of the colorectal cancer patients harbor this genotype. It was estimated that individuals carrying the CC genotype of rs3752651 had about 11-fold lower risk of developing colorectal cancer compared to those with the TT genotype (OR = 0.090, *χ*^2^ = 5.03, *p*=0.02493). Furthermore, the rs1136201 variant in the HER2 gene revealed a significant protective association with CRC. Individuals with the minor allele G of rs1136201 were 80% less likely to develop CRC compared to those having A allele (OR = 0.555, *χ*^2^ = 4.45, *p*=0.03486). The other two SNPs, rs763317 (HER1) and rs1058808 (HER2), did not show a significant association with the predisposition of colorectal cancer in the overall analysis (Table 2).

### 3.2. Association of HER1 and HER2 SNPs with Colorectal Cancer Based on the Age of Onset of the Disease

To determine whether HER1 SNPs s763317 and rs3752651 and HER2 SNPs rs1058808 and rs1136201 have any association with the age at colorectal cancer diagnosis, colorectal cancer patients were stratified based on the median age at the time of disease diagnosis as ≤58 and > 58 years, and the genotype and allele frequencies were compared to the age-matched controls. Our data demonstrated that none of the analyzed SNPs had a noticeable association with colorectal cancer suggesting that these variants do not influence early or late onset of the disease (Table 3).

### 3.3. HER1 and HER2 SNPs' Association with Colorectal Cancer Risk Based on Gender

We investigated whether gender plays any role in the association of HER1 SNPs, rs3752651 and rs763317, and HER2 SNPs, rs1058808 and rs1136201, with CRC. Our data indicated that the CC genotype of rs3752651 imparts significant protection against CRC only in men (OR = 0.077, *χ*^2^ = 5.60, *p*=0.01798). Additionally, both SNPs examined in HER2 showed a significant association with CRC but only in women. The rs1058808 increased the risk while rs1136201 was protectively associated with CRC. For the rs1058808 loci, CC homozygotic women showed approximately 3.8-fold higher risk of developing colorectal cancer compared to those having the GG genotype (OR = 3.875, *χ*^2^ = 5.65, *p*=0.01746). According to the allelic model as well, the minor allele C of rs1058808 conferred a significant association with CRC (OR = 1.665, *χ*^2^ = 4.04, *p*=0.04439). The minor allele G of rs1136201 SNP in HER2 lowers the susceptibility of developing colorectal cancer in women about 4-fold compared with those having A allele (OR = 0.244, *χ*^2^ = 7.26, *p*=0.00705). This association persisted even after Bonferroni correction for multiple comparisons. Moreover, the AG heterozygous women for this SNP also had significant protection against this cancer compared to those with the AA genotype (OR = 0.275, *χ*^2^ = 5.33, *p*=0.02093) (Table 4).

## 4. Discussion

The HER family of receptors are transmembrane tyrosine kinases; they play a key role in the regulation of various cellular functions in response to extracellular signaling. The basic aspect of signaling in the HER family is the interaction of two receptors [21]. When HER family members dimerize, it leads to transphosphorylation of their intracellular domain generating the initial signal that activates several downstream signaling pathways [21]. HER family signaling is deregulated in several cancer types, mainly in breast and lung cancers [11–13].

In the present study, we evaluated the role of SNPs rs763317 and rs3752651 residing in HER1 and rs1058808 and rs1136201 in HER2 genes in colorectal cancer in a Saudi population. Very few studies have examined the association of these SNPs with colorectal cancers. To the best of our knowledge, this is the first study to evaluate the association of HER1 and HER2 gene polymorphism with colorectal cancer risk in a Saudi Arabian population. We have demonstrated that the HER1 SNP rs3752651 and HER2 exonic SNP rs1136201 are protectively associated with colorectal cancer in the overall analysis. Additionally, gender segregation of the population revealed that the association with CRC was statistically significant in men for rs3752651 and women for rs1136201. While the CC genotype of rs3752651 provided significantly increased protection (OR = 0.077, *p*=0.01798) relative to the TT genotype in men, such association was not observed in women. Contrary to our finding of protective association in the overall analysis (OR = 0.090, *p*=0.024), Poole et al. demonstrated that the CC genotype of rs3752651 conferred 40% increased risk of colon cancer relative to those having the TT genotype. However, this association was not statistically significant for rectal cancer as well as when the cases were pooled together as colorectal cancers [10]. In glioma patients, the CC genotype of rs3752651 was found to be significantly associated with decreased overall and progression-free survival compared to the TT genotype [22].

Contradictory results pertaining to the association of rs1136201 in HER2 with various malignancies exist. While in the overall analysis of our population, the genotypes of rs1136201 were not associated with colorectal cancers, we observed a protective association with the G allele conferring 1.8-fold lower risk of developing the disease relative to the A allele. Similar to our findings, the genotypic analysis of rs1136201 in the Indian as well as Scottish Caucasian population did not show a significant correlation with colorectal carcinoma [17, 18]. Interestingly, when our population were segregated based on gender, the rs1136201 demonstrated that women carriers of the AG genotype and the G allele were at 3.6-fold (OR = 0.275, *p*=0.02093) and 4.1-fold (OR = 0.244, *p*=0.00705) lower risk of colorectal malignancy relative to the AA genotype and A allele, respectively. Similar to our findings, de Almeida et al. reported a negative association in a dominant model of analysis comparing AG + GG versus AA as well as G allele with breast cancer in a Brazilian population [23]. Additionally, Nelson et al. in a United States population from Wisconsin suggested an inverse relationship of the GG genotype of rs1136201 with invasive breast cancer. This inverse association was stronger in women aged more than 55 years, those without a family history of breast cancer, postmenopausal women with higher body mass index, and cases with regional or distant metastasis [24]. Few investigations tried to address the functional consequence of A or G allele of rs1136201 with HER2 protein overexpression. Millikan et al. found that breast tumors from women that possess the GG genotype displayed higher expression of HER2, but the results did not reach statistical significance [14]. In ovarian cancer patients from a South Indian state of Tamil Nadu, Shanmughapriya et al. demonstrated that the GG homozygotes of rs1136201 strongly expressed HER2 protein [15]. A similar association of the GG genotype and HER2 protein overexpression as well as patient survival was not observed in colorectal cancer patients [18].

Besides, HER2 gene SNP rs1058808 which did not show any association in the overall comparison demonstrated that women harboring the CC genotype were at 3.8-fold increased risk of developing colorectal cancer compared to those with the GG genotype. The rs1058808 (c.3418 C > G), also known as P1170A, is a missense variant located in exon 27 of the HER2 gene [12, 19]. Since rs1058808 and rs1136201 variants are in the coding region, they might affect the tyrosine kinase activity or structure of the protein making them unstable. HER2 gene nsSNP rs1058808 has been reported to show a negative change in Gibb's free energy; ΔΔG value more than 1 which is −1.07 is considered to be less stable by the I-Mutant 2.0 server. The Pro1170Ala change of rs1058808 is a change from polar to nonpolar amino acid that has been reported to be deleterious according to the SIFT program [25].

Similar to our overall analysis, Han et al. did not find any correlation with genotypes of rs1058808 and rs1136201 and the occurrence of breast cancer in Korean women. However, a haplotype comprising these two nonsynonymous tagging SNPs was linked with higher HER2 expression and significantly poorer prognosis [19]. Su et al. also reported a significant association between rs1058808 and HER2 protein expression. They found that patients with CG and GG genotypes of rs1058808 demonstrated higher HER2 protein expression relative to those having the CC genotype (*p* < 0.01) [26]. The mechanism related to the higher HER2 protein expression with genetic variants needs further investigations. Conflicting reports pertaining to the association of rs1058808 and rs1136201 with different cancer types have been reported [11, 13, 27–29]. In view of the involvement of HER1 and HER2 in several malignancies such as breast cancer, ovarian cancer, non-small-cell lung cancer, head and neck cancers, gastric cancer, and hepatocellular carcinoma, it would be interesting to examine the association of SNPs tested in this study with these cancers [30, 31].

Despite significant findings, the drawback of our study is that the sample size is relatively small and from a single hospital in the capital city Riyadh which receives patients from all over the country but may not represent the entire Saudi population. Additionally, since consanguinity is common in Saudi Arabia, it may have introduced some bias in our results. Another limitation of our study is that we took a simplistic view and performed an association of individual SNP with CRC; however, haplotype linkage disequilibrium analysis together with additional SNPs on same chromosome might provide broader insights into the role of HER1 and HER2 variants in the causation of the disease.

In conclusion, our preliminary analysis on selected SNPs in HER1 and HER2 genes suggests a statistically significant association of rs3752651, rs1058808, and rs1136201 with colorectal cancer in a Saudi population. These associations were gender specific and not related to the age of onset of the disease. The rs3752651 in men and rs1136201 in women showed protective association, while rs1058808 increases the risk of colorectal cancers in women. Although our results are significant, due to the small sample size, it should be validated in a larger cohort of diverse ethnicities along with haplotype linkage analysis before utilizing these SNPs as screening markers for colorectal cancers.

## Figures and Tables

**Table 1:** Test for deviation from Hardy–Weinberg equilibrium.

SNP ID	Genotype	Controls, *n* (frequency)	HWE *p* value	Cancer, *n* (frequency)	HWE *p* value
rs763317	AA	43 (0.29)	0.146801	40 (0.29)	0.538722
	GA	82 (0.55)		73 (0.52)	
	GG	24 (0.16)		27 (0.19)	

*rs3752651*	TT	119 (0.73)	0.191444	102 (0.71)	**0.045373**
	CT	38 (0.23)		41 (0.29)	
	CC	6 (0.04)		0 (0)	

*rs1058808*	GG	62 (0.39)	0.208318	44 (0.32)	0.610697
	CG	81 (0.51)		71 (0.51)	
	CC	17 (0.11)		24 (0.17)	

*rs1136201*	AA	123 (0.76)	0.672787	120 (0.85)	0.339372
	AG	37 (0.23)		21 (0.15)	
	GG	2 (0.01)		0 (0)	

HWE, Hardy–Weinberg equilibrium.

**Table 2:** HER1 and HER2 receptor SNPs' genotype and allele frequencies in colorectal cancer cases and the control population.

SNP ID	Genotype	Controls, *n* (frequency)	Colorectal cancer, *n* (frequency)	OR (95% CI)	*χ* ^2^ value	*p* ^*∗*^ value
*rs763317 HER1*	AA	43 (0.29)	40 (0.29)	Ref		
	GA	82 (0.55)	73 (0.52)	0.957 (0.561–1.632)	0.03	0.87181
	GG	24 (0.16)	27 (0.19)	1.209 (0.602–2.431)	0.28	0.59350
	**Allele**					
	A	168 (0.56)	153 (0.55)	Ref		
	G	130 (0.44)	127 (0.45)	1.073 (0.773–1.490)	0.18	0.67521

*rs3752651 HER1*	TT	119 (0.73)	102 (0.71)	Ref		
	CT	38 (0.23)	41 (0.29)	1.259 (0.752–2.106)	0.77	0.38023
	CC	6 (0.04)	0 (0)	0.090 (0.005–1.611)	5.03	**0.02493**
	**Allele**					
	T	276 (0.85)	245 (0.86)	Ref		
	C	50 (0.15)	41 (0.14)	0.924 (0.591–1.445)	0.12	0.72819

*rs1058808 HER2*	GG	62 (0.39)	44 (0.32)	Ref		
	CG	81 (0.51)	71 (0.51)	1.235 (0.749–2.038)	0.68	0.40828
	CC	17 (0.11)	24 (0.17)	1.989 (0.957–4.135)	3.45	0.06333
	**Allele**					
	G	205 (0.64)	159 (0.57)	Ref		
	C	115 (0.36)	119 (0.43)	1.334 (0.960–1.855)	2.95	0.08608

*rs1136201 HER2*	AA	123 (0.76)	120 (0.85)	Ref		
	AG	37 (0.23)	21 (0.15)	0.582 (0.322–1.051)	3.26	0.07079
	GG	2 (0.01)	0 (0)	0.205 (0.010–4.314)	1.94	0.16413
	**Allele**					
	A	283 (0.87)	261 (0.93)	Ref		
	G	41 (0.13)	21 (0.07)	0.555 (0.320–0.965)	4.45	**0.03486**

OR 95% CI, odds ratio and 95% confidence interval. ^*∗*^*p* < 0.05 was considered significant and are depicted in bold.

**Table 3:** HER1 and HER2 SNPs' genotype and allele frequencies in colorectal cancer cases and the control population based on age.

SNP ID	Genotype	Controls *n* (frequency)	Colorectal cancer *n* (frequency)	OR (95% CI)	*χ* ^2^ value	*p* ^∗^ value
**≤58**						
*rs763317 HER1*	AA	22 (0.29)	16 (0.23)	Ref		
	GA	46 (0.6)	41 (0.58)	1.226 (0.568-2.645)	0.27	0.60413
	GG	9 (0.12)	14 (0.2)	2.139 (0.744-6.151)	2.02	0.15540
	**Allele**					
	A	90 (0.58)	73 (0.51)	Ref		
	G	64 (0.42)	69 (0.49)	1.329 (0.840-2.104)	1.48	0.22426

*rs3752651 HER1*	TT	60 (0.71)	53 (0.74)	Ref		
	CT	20 (0.24)	19 (0.26)	1.075 (0.519-2.228)	0.04	0.84480
	CC	4 (0.05)	0 (0)	0.126 (0.007-2.388)	3.43	0.06403
	**Allele**					
	T	140 (0.83)	125 (0.87)	Ref		
	C	28 (0.17)	19 (0.13)	0.760 (0.405-1.428)	0.73	0.39268

*rs1058808 HER2*	GG	30 (0.36)	22 (0.31)	Ref		
	CG	44 (0.53)	32 (0.46)	0.992 (0.486-2.026)	0.00	0.98183
	CC	9 (0.11)	16 (0.23)	2.424 (0.906-6.490)	3.18	0.07462
	**Allele**					
	G	104 (0.63)	76 (0.54)	Ref		
	C	62 (0.37)	64 (0.46)	1.413 (0.894-2.232)	2.19	0.13855

*rs1136201 HER2*	AA	65 (0.78)	62 (0.87)	Ref		
	AG	17 (0.2)	9 (0.13)	0.555 (0.230-1.338)	1.75	0.18580
	GG	1 (0.01)	0 (0)	0.349 (0.014-8.737)	0.95	0.33054
	Allele					
	A	147 (0.89)	133 (0.94)	Ref		
	G	19 (0.11)	9 (0.06)	0.524 (0.229-1.197)	2.42	0.12010

**>58**						
*rs763317 HER1*	AA	21 (0.29)	24 (0.35)	Ref		
	GA	36 (0.5)	32 (0.46)	0.778 (0.366-1.654)	0.43	0.51372
	GG	15 (0.21)	13 (0.19)	0.758 (0.295-1.953)	0.33	0.56612
	**Allele**					
	A	78 (0.54)	80 (0.58)	Ref		
	G	66 (0.46)	58 (0.42)	0.857 (0.535-1.372)	0.41	0.51996

*rs3752651 HER1*	TT	59 (0.75)	49 (0.69)	Ref		
	CT	18 (0.23)	22 (0.31)	1.472 (0.710-3.051)	1.08	0.29770
	CC	2 (0.03)	0 (0)	0.240 (0.011-5.126)	1.64	0.20083
	**Allele**					
	T	136 (0.86)	120 (0.85)	Ref		
	C	22 (0.14)	22 (0.15)	1.133 (0.598–2.149)	0.15	0.70134

*rs1058808 HER2*	GG	32 (0.42)	22 (0.32)	Ref		
	CG	37 (0.48)	39 (0.57)	1.533 (0.758–3.103)	1.42	0.23381
	CC	8 (0.1)	8 (0.12)	1.455 (0.474–4.459)	0.43	0.51096
	**Allele**					
	G	101 (0.66)	83 (0.6)	Ref		
	C	53 (0.34)	55 (0.4)	1.263 (0.784–2.033)	0.92	0.33644

*rs1136201 HER2*	AA	58 (0.73)	58 (0.83)	Ref		
	AG	20 (0.25)	12 (0.17)	0.600 (0.269–1.339)	1.57	0.20989
	GG	1 (0.01)	0 (0)	0.333 (0.013–8.352)	0.99	0.31937
	Allele					
	A	136 (0.88)	128 (0.91)	Ref		
	G	22 (0.14)	12 (0.09)	0.580 (0.276–1.219)	2.10	0.14691

OR 95% CI, odds ratio and 95% confidence interval. ^*∗*^*p* < 0.05 was considered significant and are depicted in bold.

**Table 4:** HER1 and HER2 SNPs' genotype and allele frequencies in colorectal cancer cases and the control population based on gender.

SNP ID	Genotype	Controls, *n* (frequency)	Colorectal cancer, *n* (frequency)	OR (95% CI)	*χ* ^2^ value	*p* ^*∗*^ value
Male						
*rs763317 HER1*	AA	24 (0.33)	21 (0.25)	Ref		
	GA	38 (0.52)	45 (0.54)	1.353 (0.654–2.802)	0.67	0.41445
	GG	11 (0.15)	18 (0.21)	1.870 (0.722–4.844)	1.68	0.19514
	**Allele**					
	A	86 (0.59)	87 (0.52)	Ref		
	G	60 (0.41)	81 (0.48)	1.334 (0.853–2.088)	1.60	0.20592

*rs3752651 HER1*	TT	51 (0.63)	60 (0.71)	Ref		
	CT	25 (0.31)	25 (0.29)	0.850 (0.436–1.658)	0.23	0.63351
	CC	5 (0.06)	0 (0)	0.077 (0.004–1.433)	5.60	**0.01798**
	**Allele**					
	T	127 (0.78)	145 (0.85)	Ref		
	C	35 (0.22)	25 (0.15)	0.626 (0.355–1.102)	2.67	0.10247

*rs1058808 HER2*	GG	31 (0.38)	28 (0.33)	Ref		
	CG	39 (0.48)	44 (0.52)	1.249 (0.640–2.437)	0.43	0.51412
	CC	11 (0.14)	12 (0.14)	1.208 (0.460–3.169)	0.15	0.70110
	**Allele**					
	G	101 (0.62)	100 (0.6)	Ref		
	C	61 (0.38)	68 (0.4)	1.126 (0.723–1.753)	0.28	0.59945

*rs1136201 HER2*	AA	61 (0.75)	67 (0.8)	Ref		
	AG	20 (0.25)	17 (0.2)	0.774 (0.372–1.612)	0.47	0.49294
	GG	0 (0)	0 (0)	0.911 (0.018–46.620)	Na	1.00000
	**Allele**					
	A	142 (0.88)	151 (0.9)	Ref		
	G	20 (0.12)	17 (0.1)	0.799 (0.403–1.587)	0.41	0.52159

Female						
*rs763317 HER1*	AA	19 (0.25)	19 (0.34)	Ref		
	GA	44 (0.58)	28 (0.5)	0.636 (0.288–1.406)	1.25	0.26263
	GG	13 (0.17)	9 (0.16)	0.692 (0.240–2.001)	0.46	0.49638
	**Allele**					
	A	82 (0.54)	66 (0.59)	Ref		
	G	70 (0.46)	46 (0.41)	0.816 (0.498–1.337)	0.65	0.42027

*rs3752651 HER1*	TT	68 (0.83)	42 (0.72)	Ref		
	CT	13 (0.16)	16 (0.28)	1.993 (0.872–4.555)	2.72	0.09881
	CC	1 (0.01)	0 (0)	0.537 (0.021–13.492)	0.61	0.43320
	**Allele**					
	T	149 (0.91)	100 (0.86)	Ref		
	C	15 (0.09)	16 (0.14)	1.589 (0.752–3.360)	1.49	0.22221

*rs1058808 HER2*	GG	31 (0.39)	16 (0.29)	Ref		
	CG	42 (0.53)	27 (0.49)	1.246 (0.575–2.699)	0.31	0.57754
	CC	6 (0.08)	12 (0.22)	3.875 (1.226–12.248)	5.65	**0.01746**
	**Allele**					
	G	104 (0.66)	59 (0.54)	Ref		
	C	54 (0.34)	51 (0.46)	1.665 (1.011–2.741)	4.04	**0.04439**

*rs1136201 HER2*	AA	62 (0.77)	53 (0.93)	Ref		
	AG	17 (0.21)	4 (0.07)	0.275 (0.087–0.869)	5.33	**0.02093**
	GG	2 (0.02)	0 (0)	0.234 (0.011–4.974)	1.69	0.19425
	**Allele**					
	A	141 (0.89)	110 (0.96)	Ref		
	G	21 (0.13)	4 (0.04)	0.244 (0.081–0.732)	7.26	**0.00705**

OR 95% CI, odds ratio and 95% confidence interval; na, not analyzable. ^*∗*^*p* < 0.05 was considered significant and are depicted in bold.

## Data Availability

All data used to support the findings of this study are included within the article.
